# Supplementary figures and images for: Structural insights into the recruitment of viral type 2 IRES to ribosomal preinitiation complex for protein synthesis
Source: eLife. 2026 Jun 25;14:RP107788. doi: 10.7554/eLife.107788 (PMC13299598; doi:10.7554/eLife.107788)

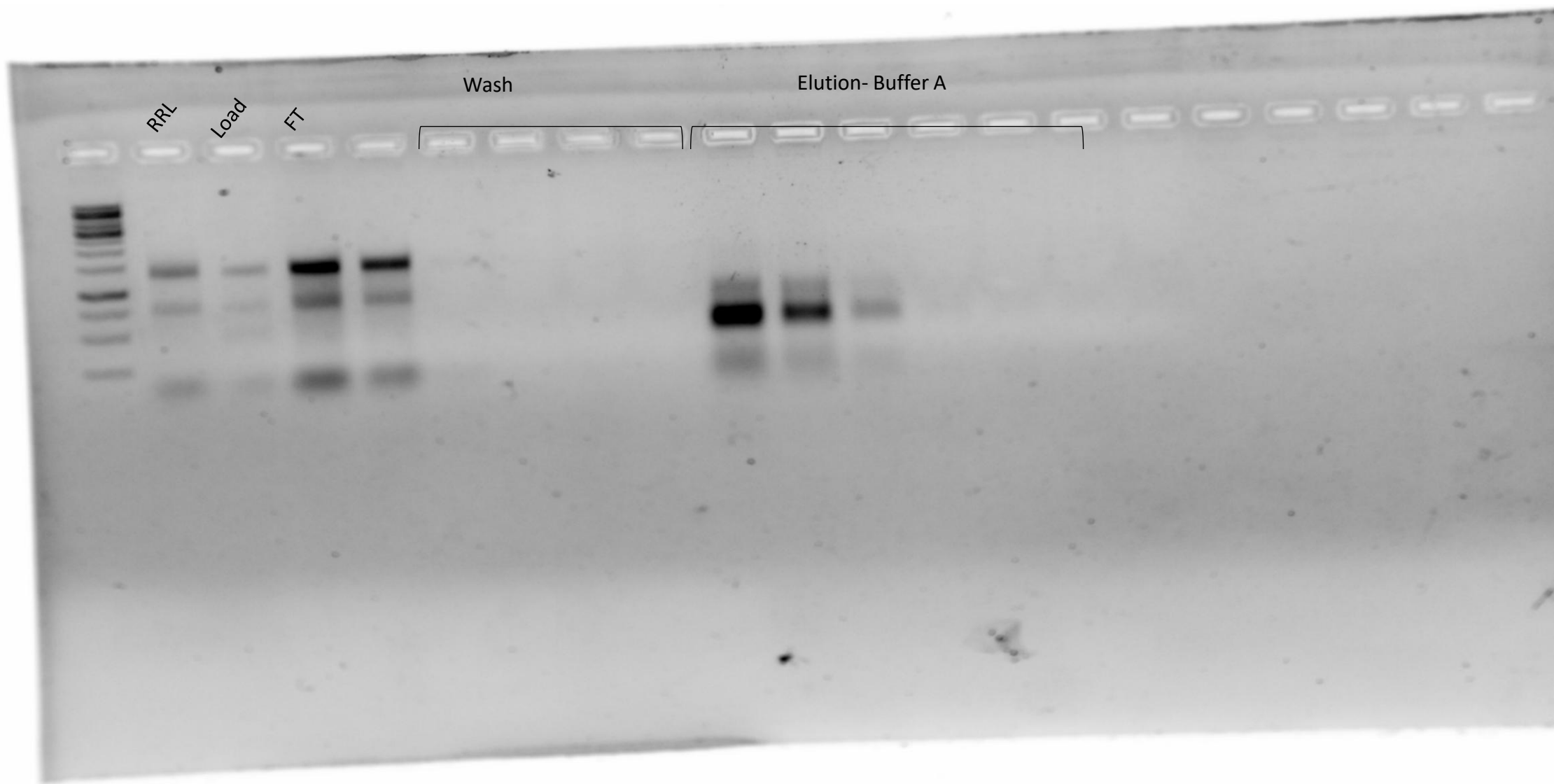

Supplement: Figure 1—figure supplement 1—source data 1. — Isolation of encephalomyocarditis virus (EMCV) internal ribosome entry site (IRES)-48S preinitiation complex (PIC) from Talon affinity chromatography, including Talon affinity chromatography profile. [file elife-107788-fig1-figsupp1-data1.zip › Source_data_labelled/Figure 1- Figure supplement 1 (A)- source data 1_labelled.pdf]

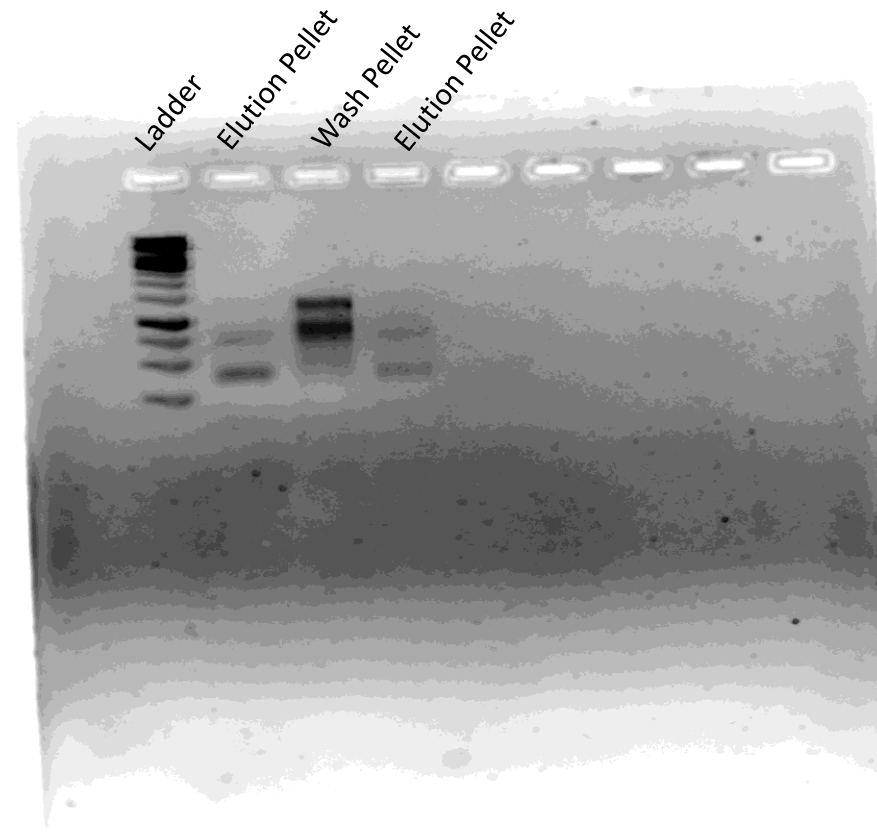

Supplement: Figure 1—figure supplement 1—source data 1. — Isolation of encephalomyocarditis virus (EMCV) internal ribosome entry site (IRES)-48S preinitiation complex (PIC) from Talon affinity chromatography, including Talon affinity chromatography profile. [file elife-107788-fig1-figsupp1-data1.zip › Source_data_labelled/Figure 1- Figure supplement 1 (A)- source data 2-labelled.pdf]

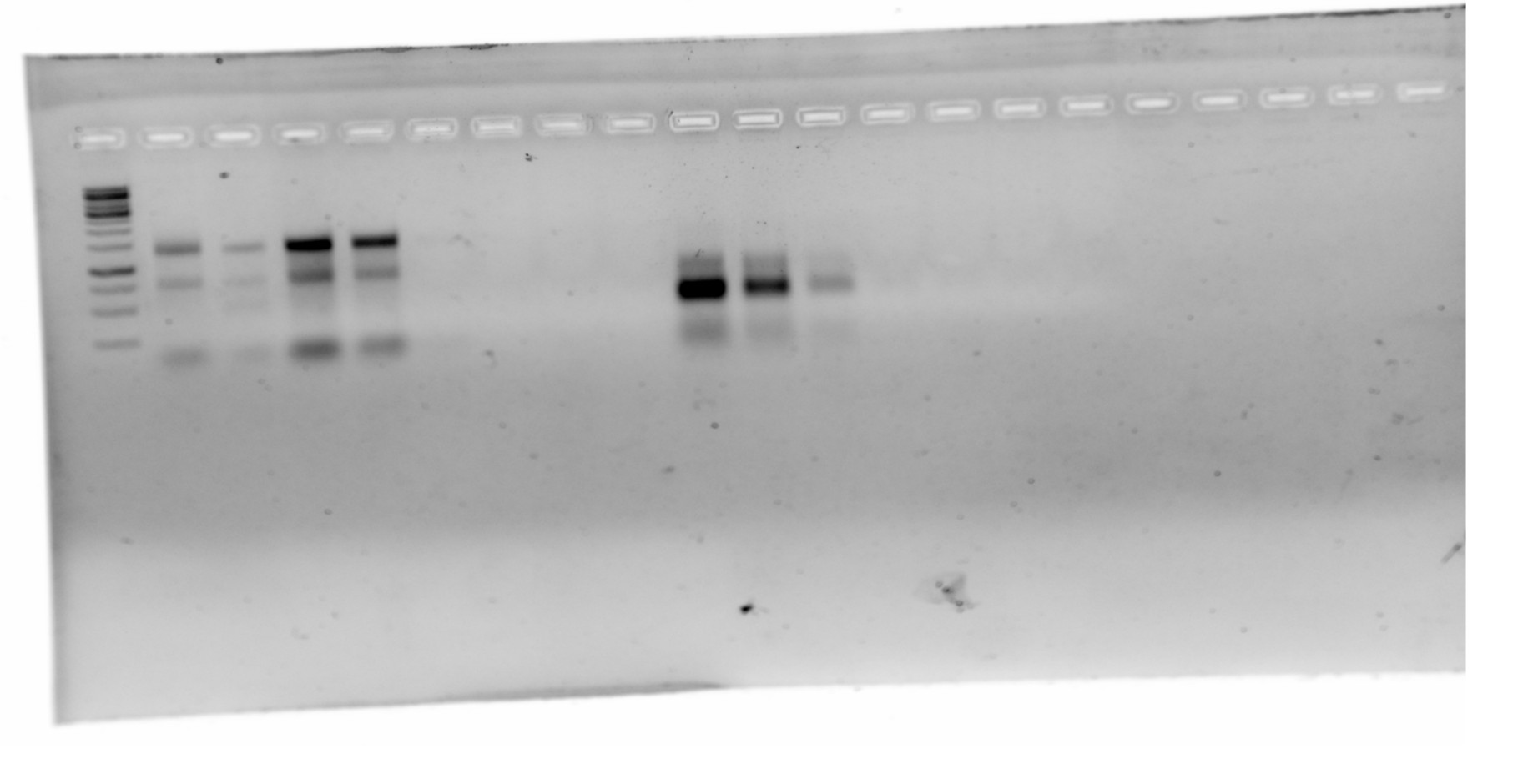

Supplement: Figure 1—figure supplement 1—source data 2. — Analysis of elution fractions – pelleted using 30% cushion on 1.5% agarose gel. [file elife-107788-fig1-figsupp1-data2.zip › Source_data_unlabelled/Figure 1- Figure supplement 1 (A)- source data 1.jpg]

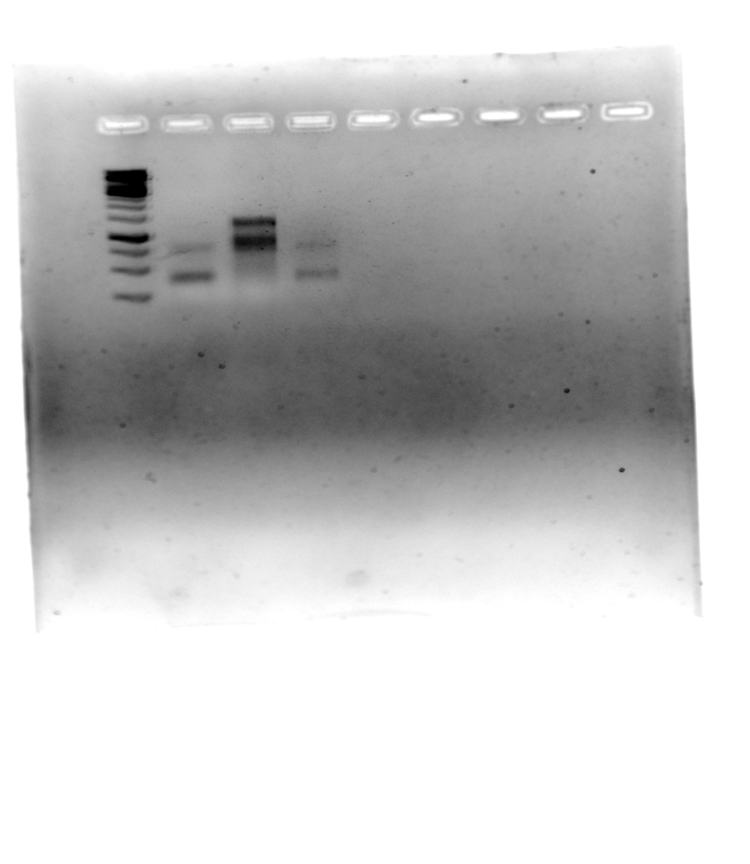

Supplement: Figure 1—figure supplement 1—source data 2. — Analysis of elution fractions – pelleted using 30% cushion on 1.5% agarose gel. [file elife-107788-fig1-figsupp1-data2.zip › Source_data_unlabelled/Figure 1- Figure supplement 1 (A)- source data 2.png]
