# Supplementary material for: Structural insights into the recruitment of viral type 2 IRES to ribosomal preinitiation complex for protein synthesis
Source: eLife. 2026 Jun 25;14:RP107788. doi: 10.7554/eLife.107788 (PMC13299598; doi:10.7554/eLife.107788)
Supplement: Supplementary file 1. [file elife-107788-supp1.docx]

**Supplementary File 1a: EMCV IRES domain sequences used as inputs for Alphafold3 prediction of IRES domain tertiary structure**

| Domain | Residues | Sequence |
| --- | --- | --- |
| D | 276-319 | CCCCCUAACGU**UACUGGCCGAAGCCGCUUGGAAUAAGGCCGGUG** |
| E | 320-336 | U**GCGUUUGUCUAUAUGU** |
| F | 337-371 | UAUUUUCCAC**CAUAUUGCCGUCUUUUGGCAAUGUG** |
| G | 372-400 | **AGGGCCCGGAAACCUGGCCCU**GUCUUCUU |
| H | 401-450 | **GACGAGCAUUCCUAGGGGUCUUUCCCCUCUCGCCAAAGGAAUGCAAGGUC** |
| I | 451-678 | **UGUUGAAUGUCGUGAAGGAAGCAGUUCCUCUGGAAGCUUCUUGAAGACAAACAACGUCUGUAGCGACCCUUUGCAGGCAGCGGAACCCCCCACCUGGCGACAGGUGCCUCUGCGGCCAAAAGCCACGUGUAUAAGAUACACCUGCAAAGGCGGCACAACCCCAGUGCCACGUUGUGAGUUGGAUAGUUGUGGAAAGAGUCAAAUGGCUCUCCUCAAGCGUAUUCAACA** |
| J-K | 679-789 | AGG**GGCUGAAGGAUGCCCAGAAGGUACCCCAUUGUAUGGGAUCUGAUCUGGGGCCUCGGUGCACAUGCUUUACAUGUGUUUAGUCGAGGUUAAAAAACGUCUAGGCC**CCCC |
| L | 790-816 | **GAACCACGGGGACGUGGUUU**UCCUUUGAAAA |

**Supplementary file 1b: List of Oligos or primers used for molecular cloning**

| Oligo name | Sequence |
| --- | --- |
| PTB1_Fwd | CGGGATCCATGGACGGCATTGTCCCAGA |
| PTB1_Rev | CCCAAGCTTCTAGATGGTGGACTTGGAG |
| PTB1_3C_Fwd | CGGGATCCCTGGAGGTGCTCTTCCAGGGCCCTGGCGGCTCCATGGACGGCATTGTCCCAG |
| PTB1_Stop_Rev | CCCAAGCTTCTAGATGGTGGACTTGGAGAAGG |
| EMCV_IRES_905-Fwd | CGGGATCCCCCCCTAACGTTACTGGCC |
| EMCV_IRES_905-Rev | GCTCTAGAGCAGAGCATTTTGGGCATTCCTCAAAAG |
| CAAAA_GCTGA_Rev | CAGGTGTATCTTATACACGTGGC**TCAGC**GCCGCAGAGGCACCTGTCGCCAG |
| GCGA_TACG_Rev | GCCGCAGAGGCACCTG**CGTA**CAGGTGGGGGGTTCCGCTGCCTGCAA |
