# Supplementary material for: Structural insights into the recruitment of viral type 2 IRES to ribosomal preinitiation complex for protein synthesis
Source: eLife. 2026 Jun 25;14:RP107788. doi: 10.7554/eLife.107788 (PMC13299598; doi:10.7554/eLife.107788)
Supplement: Peer review history

# Editor report: eLife Assessment

**DOI:** 10.7554/eLife.107788.3.sa0

**Authors:** Yogesh K Gupta

**Affiliations:** The University of Texas Health Science Center at San Antonio United States

**Keywords:** Solid, Valuable

This manuscript offers **valuable** structural and mechanistic insights into the assembly of the Type II internal ribosome entry site (IRES) from encephalomyocarditis virus (EMCV) and the translation initiation complex, revealing a direct interaction between the IRES and the 40S ribosomal subunit. A **solid** experimental strategy, combining cryo-EM analysis, complementary biochemistry, and detailed structural comparisons, provides mechanistic insights into IRES-based translation initiation systems. This paper will attract researchers in cap-independent translation, host-pathogen interactions, and virology.

# Referee report: Reviewer #1 (Public review)

**DOI:** 10.7554/eLife.107788.3.sa1

Summary:

The authors have studied how a virus (EMCV) uses its RNA (Type 2 IRES) to hijack the host's protein-making machinery. They use cryo-EM to extract structural information about the recruitment of viral Type 2 IRES to ribosomal pre-IC. The authors propose a novel interaction mechanism in which the EMCV Type 2 IRES mimics 28S rRNA and interacts with ribosomal proteins and initiator tRNA (tRNAi).

Strengths:

(1) Getting structural insights about the Type 2 IRES-based initiation is novel.

(2) The study allows a good comparison of other IRES-based initiation systems.

(3) The manuscript is well-written and clearly explains the background, methods, and results.

Comments on revised version:

I have gone through the revised manuscript by Das and Hussain along with the rebuttal comments. While the poor resolution of the ribosomal complex limits detailed analysis of the molecular interactions, addition of the luciferase reporter assay in the supplementary has enriched the paper.

# Referee report: Reviewer #2 (Public review)

**DOI:** 10.7554/eLife.107788.3.sa2

Summary:

The field of protein translation has long sought the structure of a Type 2 Internal Ribosome Entry Site (IRES). In this work, Das and Hussain pair cryo-EM with algorithmic RNA structure prediction to present a structure of the Type 2 IRES found in Encephalomyocarditis virus (EMCV). Using medium to low resolution cryo-EM maps, they resolve the overall shape of a critical domain of this Type 2 IRES. They use algorithmic RNA prediction to model this domain onto their maps and attempt to explain previous results using this model.

Strengths:

(1) This study reveals a previously unknown/unseen binding modality used by IRESes: a direct interaction of the IRES with the initiator tRNA.

(2) Use of an IRES-associated factor to assemble and pull down an IRES bound to the small subunit of the ribosome from cellular extracts is innovative.

(3) Algorithmic modeling of RNA structure to complement medium to low resolution cryo-EM maps, as employed here, can be implemented for other RNA structures.

Comments on revised version:

Thanks to the authors for providing thorough responses to the reviewer questions and comments. I appreciate their attempts of improving overall resolution of the complex via various processing strategies that the reviewers suggested.

The authors interpretations of their cryo-EM data match those reported by Bhattacharjee et al. 2025 (EMCV-IRES 48S) and can be contextualized in the light of Velazquez et al. 2025 (poliovirus IRES-48S).

The authors' contextualization of their results with previously published studies (Discussion section lines 355-402) is satisfactory to me but can be improved.

# Referee report: Reviewer #3 (Public review)

**DOI:** 10.7554/eLife.107788.3.sa3

Summary:

Type II IRES, such as those from encephalomyocarditis virus (EMCV) and foot-and-mouth disease virus (FMDV), mediate cap-independent translation initiation by using the full complement of eukaryotic initiation factors (eIFs), except the cap-binding protein eIF4E. The molecular details of how IRES type II interacts with the ribosome and initiation factors to promote recruitment have remained unclear. Das and Hussain used cryo-electron microscopy to determine the structure of a translation initiation complex assembled on the EMCV IRES. The structure reveals a direct interaction between the IRES and the 40S ribosomal subunit, offering mechanistic insight into how type II IRES elements recruit the ribosome.

Strengths:

The structure reveals a direct interaction between the IRES and the 40S ribosomal subunit, offering mechanistic insight into how type II IRES elements recruit the ribosome.

Comments on revised version:

The revised manuscript does not improve the resolution; however, the authors provide a detailed and well-reasoned rationale that directly addresses the concerns I raised about their structural interpretation. In addition, two independent preprints have been released since the initial submission. In one case, the authors report a higher-resolution, and importantly, all three studies present consistent assignments and interpretations. Together, these observations strengthen confidence in the authors' conclusions. I therefore do not have major concerns regarding the publication of this revised manuscript.

# Author comment: Author response

**DOI:** 10.7554/eLife.107788.3.sa4

**Authors:** Deepakash Das, Tanweer Hussain

**Affiliations:** Indian Institute of Science Bangalore Bangalore India; Indian Institute of Science Bangalore Bangalore India

The following is the authors’ response to the original reviews.

**eLife Assessment**

This manuscript offers valuable structural and mechanistic insights into the structure and assembly of the Type II internal ribosome entry site (IRES) from encephalomyocarditis virus (EMCV) and the translation initiation complex, revealing a direct interaction between the IRES and the 40S ribosomal subunit. While a solid cryo-EM method was used, enhancing the overall resolution or adding complementary biochemical data would further improve the clarity and impact of this study. This manuscript will attract researchers in cap-independent translation, host-pathogen interactions, and virology.

We thank the editorial team for a favourable assessment and for mentioning our work as ‘valuable’. In the following sections, we have addressed the weaknesses and recommendations pointed out by the Reviewers and hope for an improvement in the description of this work.

**Public Reviews:**

**Reviewer #1 (Public review):**

Summary:

The authors have studied how a virus (EMCV) uses its RNA (Type 2 IRES) to hijack the host's protein-making machinery. They use cryo-EM to extract structural information about the recruitment of viral Type 2 IRES to ribosomal pre-IC. The authors propose a novel interaction mechanism in which the EMCV Type 2 IRES mimics 28S rRNA and interacts with ribosomal proteins and initiator tRNA (tRNAi).

Strengths:

(1) Getting structural insights about the Type 2 IRES-based initiation is novel.

(2) The study allows a good comparison of other IRES-based initiation systems.

(3) The manuscript is well-written and clearly explains the background, methods, and results.

We thank Reviewer 1 for appreciating our efforts and finding structural insights about the Type 2 IRES-based initiation presented in this study as novel.

Weaknesses:

(1) The main weakness of the work is the low resolution of the structure. This limits the possibility of data interpretation at the molecular level.

However, despite the moderate resolution of the cryo-EM reconstructions, the model fits well into the density. The analysis of the EMCV IRES-48S PIC structure is thorough and includes meaningful comparisons to previously published structures (e.g., PDB IDs - 7QP6 and 7QP7). These comparisons showed that Map B1 represents a closed conformation, in contrast to Map A in the open state (Figure 2). Additionally, the proposed 28S rRNA mimicry strategy supported by structural superposition with the 80S ribosome and sequence similarity between the I domain of the IRES and the h38 region of 28S rRNA (Fig. 4) is well-justified.

We agree that the low resolution of the map has compromised the data interpretation at the molecular level, and we thank the reviewer for appreciating our findings at this resolution. Due to the low resolution, we have reported findings for stretches or regions such as the domain I loops and stems, rather than individual nucleotides.

(2) The lack of experimental validation of the functional importance of regions like the GNRA and RAAA loops is another limitation of this study.

We agree about the lack of additional experiments other than Cryo-EM for probing the importance of regions such as GNRA and RAAA loops in this study. Previously, multiple studies have reported on the importance of GNRA and RAAA loops and we have cited them in the manuscript. The essentiality of RAAA loop for type 2 IRES was demonstrated in earlier report López de Quinto and Martínez-Salas, 1997 (Cited in manuscript). Further, the conservation of this loop across the type 2 IRES family adds to the importance of this loop (Manuscript Figure 6B). This loop and its flanking G-C stem are similar to h38 of 28S rRNA, and it appears that RAAA loop adopts a mimicry mechanism to interact with the 40S ribosomal protein- uS19, thus highlighting its importance for interaction with 40S. Experiments destabilising the G-C stem also compromise IRES activity, as shown for the case of FMDV IRES (Fernández et al 2011). Previous studies related to the mutation of the GNRA or GCGA loop in EMCV IRES have shown a deficiency in IRES activity (Roberts and Belsham, 1997; Robertson et al 1999), suggesting the importance of these regions in the viral IRES biology, and these reports are cited in the manuscript. Not only EMCV IRES, but mutation in the GUAA (representative of GNRA) loop of FMDV IRES also showed a significant reduction in IRES activity (López de Quinto and Martínez-Salas, 1997). In this work, we observe that the GCGA loop interacts with tRNA_i_ in the EMCV IRES-48S PIC, thus implicating the importance of this loop. Moreover, incubation of FMDV IRES with 40S ribosomes has shown a decrease in SHAPE reactivity in domain 3 apex (position 170- 200 nucleotides) (Lozano et al 2018), which corresponds to EMCV IRES domain I apex.

However, to address this concern in the revised manuscript we mutated these loops and performed luciferase assay (Supplementary figure 4 A). The results showed decreased IRES activity (Pg 10) and correlated with previous reports demonstrating the importance of these regions for overall IRES activity.

(3) Minor modifications related to data processing and biochemical studies will further validate and strengthen the findings.

(a) In the cryo-EM data section, the authors should include an image showing rejected particles during 2D classification. This would help readers understand why, despite having over 22k micrographs with sufficient particle distribution and good contrast, only a smaller number of particles were used in the final reconstruction. Additionally, employing map-sharpening tools such as Ewald sphere correction, Bayesian polishing, or reference-based motion correction might further improve the quality of the maps. Targeting high-resolution structures would be particularly informative.

We have included the image for rejected 2D classes (Author response image 1). We agree with the Reviewer’s query related to the huge number of micrographs and relatively smaller number of particles for the final reconstruction. Since the total number of micrographs (22000) is the summation of multiple datasets, prepared and collected at different times, the distribution of the particles per micrograph was not uniform in all sessions, ranging from good to poor. Among these, around 8000 micrographs have poor particle number and distribution. As a result, the number of particles per micrograph is heterogeneous across the compiled dataset, and only 237054 ribosomal particles were obtained after multiple rounds of 2D and 3D classification. Further, the final reconstruction was performed using particles obtained after masked classification for IRES and ternary complex density. Only the particles that show the best density for both IRES and ternary complex are used for this map. Another set of particles that have only a portion of IRES and NO density for ternary complex forms another map. And we have a third map with an empty 40S.

We thank the reviewer for the suggestions to improve the quality of the maps further. As suggested, we started with the processing of the data. However, during this process the common computational cluster that were using for this data processing had to be physically relocated, and unfortunately after the relocation we faced technical issues in accessing and continuing with the processing. Several attempts to resolve the issue with the help of IT team failed. Thus, we lost 3-4 months without any progress. Therefore, we used Relion on our in-house workstation to process the data files from the start, as our in-house computational resources are unequipped to run cryoSPARC processes (for large dataset due to memory limitations).

We reprocessed the datasets in Relion5 and did ‘Bayesian Processing’, for reference-based beam-induced motion correction per-particle. Post-processing, we used cryoSPARC to merge the particles and tried classifying the good ribosome particles using focus-based masked classification, as shown in Supplementary Figure 1.1. However, this processing did not improve the resolution, as Map B (containing 40S, tRNA, IRES) had an overall resolution of 4.8 Å (Author response image 2). Therefore, we would like to report the same maps as given in the initial submission.

We estimated the time to redo the entire processing using cryoSPARC on the common computational cluster, and it would take us another 3-4 months or more and we do not anticipate a massive improvement in the extra density.

**Author response image 1.** The selected 2D classes and the rejected 2D classes from initial round of classification, and the final selected 2D classes, which were subjected to Ab-initio reconstruction to get the good ribosome particles.

**Author response image 2.** Reprocessing of the entire dataset using Relion5 for polishing of selected particles, followed by 3D classification and refinements in cryoSPARC.

(b) The strategic modelling of different IRES domains into the density, particularly the domain into the region above the 40S head, is appreciable. However, providing the full RNA tertiary structure (RNAfold) of the EMCV IRES (nucleotides 280-905) would better explain the logic behind the model building and its molecular interpretation.

We thank the reviewer for appreciating the modelling of the domain I apex in the cryo-EM density. We tried to predict the full tertiary structure of the IRES using Alphafold3; however, inclusion of the full-length sequence from 280-905 gave models of extremely low confidence (Author response image 3), and a few domains do not abide by the secondary structure of EMCV IRES as reported in Duke et al 1992.

**Author response image 3.** Prediction of tertiary structure of EMCV IRES (280-905 nucleotides) and zoomed features for each domain present in the IRES. The predicted aligned error plot for the RNA structure is shown.

We used individual domains of EMCV IRES and predicted the tertiary structure, independent of other IRES domain using Alphafold3. As a result, the confidence scores improved, and the tertiary structures also correlated with the experimentally determined EMCV IRES secondary structure (Duke et al 1992; Maloney and Joseph, 2024). Although the overall tertiary structure of EMCV IRES is lacking, recent studies were able to solve the structures of EMCV IRES domains in complex with their respective binding proteins. We superimposed the independently predicted domains D, E, and F tertiary structure on the NMR ensemble of IRES domain D to F with PTB1 (Dorn et al 2023), where the predicted domains fit in the experimental model. Similarly, we used the cryo-EM structure of domain J-K-eIF4G-eIF4A (Imai et al 2023) and found a close fit with the predicted structures. The analysis highlighted that the domain I apex serves as the best fit with the extra density with respect to architecture and fitting. This analysis is now added in the revised manuscript in Supplementary figure- 3.2.

Furthermore, 3D structural models of FMDV IRES domains 2, 3, and 4 (corresponding to EMCV IRES domains- H, I, and J-K) were predicted from SHAPE reactivity values and RNAComposer server (Figure 3, Lozano et al 2018). The predicted architecture of domain 3 apex (FMDV IRES) coincides with our domain I apex model (EMCV IRES).

(c) Although the authors compare their findings with other types of IRESs (Types 1, 3, and 4), there is no experimental validation of the functional importance of regions like the GNRA and RAAA loops. Including luciferase-based assays or mutational studies of these regions for validation of structural interpretations is strongly recommended.

We have discussed the possibility of how the other IRESs, such as type 1 and type 5, might use similar strategies as EMCV IRES to assemble the 48S PIC, given the similarity in the motif sequence and position across the viral IRESs. Like EMCV IRES, the type 1 IRES (Poliovirus, Coxsackie virus, etc.) also harbours the GNRA loop, preceded by a C-rich loop at its longest domain, known for long-range RNA-RNA interactions. The segment harbouring GNRA loop is highly conserved across the type 1 family of IRESs (Kim et al 2015). The Aichi viral IRES harbours a GNRA loop in its longest domain, that is, domain J. Deletion of the GNRA loop has compromised the IRES activity; however, substitution mutations in this region have elevated the IRES activity or remained unaltered (Yu et al 2011). We have hypothesized that these IRESs might use the GNRA motifs in their longest domain (domain IV in type 1, and domain J in Aichi virus- type 5) based on the location and architecture to that of EMCV IRES, where GNRA is present in the longest domain (I) and preceded by a C-rich loop where it can potentially mediate long-range interactions with tRNA_i_, as all these IRESs require eIF2-ternary complex for the formation of 48S PIC. Parallelly, like EMCV IRES, type 1 and type 5 IRESs have the placement of this GNRA motif-containing domain before the eIF4G-binding domain. Thus, we suggest the possibility of adoption of a similar strategy by these IRESs to interact with tRNA_i_ during the formation of 48S PIC. During the revision of this work a preprint reported the structure of polioviral IRES-48S PIC where domain IV apex (similar to domain I apex in EMCV IRES) interacts with uS13 and uS19, and the GNRA loop directly interacts with tRNA_i_ during start codon recognition (Velazquez et al 2025). We hypothesize that Aichiviral IRES might use this motif to mediate long-range interactions with tRNA_i_, similar to type 1 and type 2 IRESs, as all these IRESs require eIF2-ternary complex for the formation of 48S PIC.

**Reviewer #2 (Public review):**

Summary:

The field of protein translation has long sought the structure of a Type 2 Internal Ribosome Entry Site (IRES). In this work, Das and Hussain pair cryo-EM with algorithmic RNA structure prediction to present a structure of the Type 2 IRES found in Encephalomyocarditis virus (EMCV). Using medium to low resolution cryo-EM maps, they resolve the overall shape of a critical domain of this Type 2 IRES. They use algorithmic RNA prediction to model this domain onto their maps and attempt to explain previous results using this model.

Strengths:

(1) This study reveals a previously unknown/unseen binding modality used by IRESes: a direct interaction of the IRES with the initiator tRNA.

(2) Use of an IRES-associated factor to assemble and pull down an IRES bound to the small subunit of the ribosome from cellular extracts is innovative.

(3) Algorithmic modeling of RNA structure to complement medium to low resolution cryo-EM maps, as employed here, can be implemented for other RNA structures.

We thank Reviewer 2 for positive and encouraging comments on our work, appreciating our ‘innovative’ approach of using IRES-associated factor to assemble and pull down the IRES-bound ribosomal complex.

Weaknesses:

(1) Maps at the resolution presented prevent unambiguous modelling of the EMCV-IRES. This, combined with the lack of any biochemical data, calls into question any inferences made at the level of individual nucleotides, such as the GNRA loop and CAAA loop (Figure 4).

We understand the concerns raised by the reviewer related to the resolution of the EMCV IRES-48S PIC map. We refrained from commenting on individual nucleotides or molecular interactions in the manuscript. Instead, we discuss loops, RNA stretches or motifs that could be inferred with more confidence in the IRES density as shown in Figure 4. The EMCV IRES can directly interact with the 40S ribosome using its domain H and I (Chamond et al 2014), however, the details of this interaction were unknown. We observe that the CAAA loop of domain I apex interacts with 40S ribosome based on the placement of a portion of domain I in the cryo-EM map. This is also reflected in the SHAPE data (Chamond et al 2014-Supplementary figures 2, and 8), where a decrease in reactivity is evident in the presence of 40S ribosome. In addition, incubation of EMCV IRES with rabbit reticulocyte lysate (RRL) offered protection to domain I apex regions, which included the CAAA loop (Maloney and Joseph, 2024- Figure 4b).

Furthermore, this decrease in SHAPE reactivity pattern is evident for FMDV IRES domain 3 apex (similar to domain I in EMCV IRES) in the presence of 40S ribosome (Lozano et al 2018). Thus, these studies are consistent with the placement of IRES model in the cryo-EM map. Moreover, we performed structural analysis (mentioned above) which showed that the domain I apex serves as the best fit with the extra density with respect to architecture and fitting (Supplementary figure- 3.2).

(2) The EMCV IRES contains an upstream AUG at position 826, where the PIC can assemble (Pestova et al 1996; PMID 8943341). It is unclear if this start codon was mutated in this study. If it were not mutated, placement of AUG-834 over AUG-826 in the P-site is unexplained.

We thank the reviewer for bringing up this point, as we missed mentioning this in the initial submission. The EMCV IRES does not require scanning and directly positions the AUG-834 at the P site (Pestova et al 1996). In Pestova et al 1996, the intensity of the toeprint at AUG-834 is more intense than that of AUG-826. Further, AUG-834 lies in the Kozak context, whereas AUG-826 has a poor Kozak context, and AUG-826 codon is not in-frame with AUG-834. Therefore, the synthesis of the polypeptide requires AUG-834 at the P site. In our cryo-EM map, we observed that the tRNA_i_ is in a P_IN_ state, which indicates the recognition of the start codon, and we reasoned that it is more likely that AUG-834 is placed at the P site than AUG-826. We have mentioned this in the revised manuscript as we had NOT mutated AUG-826 (Pg 8).

(3) The claims the authors make about (i) the general overall shape and binding site of the IRES, (ii) its gross interaction with the two ribosomal proteins, (iii) the P-in state of the 48S, (iv) the rearrangement of the ternary complex are all warranted. Their claims about individual nucleotides or smaller stretches of the IRES-without any supporting biochemical data-is not warranted by the data.

We thank the reviewer for warranting major claims, and due to the low-resolution we have reported findings for stretches or regions such as the domain I loops and stems, rather than individual nucleotides. The interaction of domain I apical region with uS13, uS19, and tRNA_i_ is also observed the high-resolution structure of reconstituted EMCV IRES-48S PIC that was reported in a preprint while our work was under peer review process (Bhattacharjee et al 2025). Thus, the reconstituted EMCV IRES-48S PIC (Bhattacharjee et al 2025) also supports our assignment of domain I and its conserved loops, interacting with ribosome and tRNA_i_.

**Reviewer #3 (Public review):**

Summary:

Type II IRES, such as those from encephalomyocarditis virus (EMCV) and foot-and-mouth disease virus (FMDV), mediate cap-independent translation initiation by using the full complement of eukaryotic initiation factors (eIFs), except the cap-binding protein eIF4E. The molecular details of how IRES type II interacts with the ribosome and initiation factors to promote recruitment have remained unclear. Das and Hussain used cryo-electron microscopy to determine the structure of a translation initiation complex assembled on the EMCV IRES. The structure reveals a direct interaction between the IRES and the 40S ribosomal subunit, offering mechanistic insight into how type II IRES elements recruit the ribosome.

Strengths:

The structure reveals a direct interaction between the IRES and the 40S ribosomal subunit, offering mechanistic insight into how type II IRES elements recruit the ribosome.

Weaknesses:

While this reviewer acknowledges the technical challenges inherent in determining the structure of such a highly flexible complex, the overall resolution remains insufficient to fully support the authors' conclusions, particularly given that cryo-EM is the sole experimental approach presented in the manuscript.

The study is biologically significant; however, the authors should improve the resolution or include complementary biochemical validation.

We thank Reviewer 3 for acknowledging the technical challenges in this study and finding our study biologically significant. We understand the concerns related to low resolution and the requirement of complementary biochemical validation for our reported observations and interpretations in the manuscript. We tried to improve the resolution, but the improvement was not sufficient to resolve the IRES at the nucleotide level. Independently, another group has reported the same findings at a higher resolution while our work was under peer review process (Bhattacharjee et al 2025), which corroborates our structural data on EMCV IRES and its interaction with ribosome and tRNA_i_ in its 48S PIC stage. Further, in the revised manuscript we also present biochemical validation for GNRA and RAAA loops in EMCV IRES. We mutated these loops and performed luciferase assay (Supplementary figure 4 A). The results showed decreased IRES activity (Pg 10) and correlated with previous reports (Roberts and Belsham, 1997; López de Quinto and Martínez-Salas, 1997; Robertson et al 1999) demonstrating the importance of these regions for overall IRES activity.

**Reviewing Editor Comments:**

The reviewers' comments are appended. While the reviewers acknowledge the complexity associated with this system, they also raised concerns about the modeling of RNA and registering its sequence in low-resolution maps. We believe that the strength of evidence and overall impact of your study can be elevated by providing higher-resolution cryo-EM data or complementary biochemical studies and addressing reviewers' concerns.

**Reviewer #2 (Recommendations for the authors):**

(1) Science:

Have the authors tried a focused refinement (local refinement in cryoSPARC) using a generous mask that encloses the head and the IRES but excludes the ternary complex and the body of the 40S? This can be done with all the particles in map B (~55K) and has the possibility of improving the resolution of domain I which can be subsequently used to build a better model of the IRES. See the middle right panel, light yellow colored mask in Figure 1A in PMID 37659578 for the type of mask being suggested.

We did another round of 2D classification to eliminate any residual junk in the ~55k particle set, corresponding to Map B. Post classification, 49439 particles were selected and refined using non-uniform refinement to get Map B11. The overall resolution of Map B11 was 4.6 Å. Thereafter, we made a mask around the 40S head-IRES-tRNA on Map B11 and subjected the class for local refinement. The overall local resolution in the masked region improved to 4.5 Å (Author response image 4).

**Author response image 4.** Data processing- Map B particles were 2D classified, and further junk was cleared as rejected particles. The selected particles were refined using non-uniform refinement to get Map B11, and later, a focused mask circling the head-tRNA-IRES region was used for local refinement in the region to yield map B111.

We estimated the local resolution across the focused region in Map B111 and compared this with that of Map B (Author response image 5). The local refinement shows minor improvement in the local resolution in this region, and is not sufficient to resolve the IRES density at the level of nucleotides.

**Author response image 5.** Comparison of local resolution across head-IRES-tRNA in map B1 (as reported in the manuscript) and Map B111.

(2) Presentation:

(a) Please use the previously established convention of naming the domains: "domain I", "domain H", etc, instead of "I domain" or "J-K domain" while describing parts of the IRES.

We have made the changes as per the established convention.

(b) Figure 2B reports a 6.9 A distance vs. 7 A in the text. Please use ~ or approximately to keep numbers consistent.

We have used ~ symbol to suggest the approximate distance.

(c) References missing on page 15 when referring to "previously determined HCV and CrPV structures".

We have added the references (Pg 12).

(d) Please edit the text for typos and sentence structure.

The typos and sentence structure were corrected wherever necessary.

(e) Some phrases and sentences (e.g. last few sentences of the first paragraph in the discussion) could be rewritten for clarity.

Previous sentence- “The domain I of EMCV IRES is similar to domain IV of polioviral IRES (or other type 1 IRESs such as Coxsackie viral IRES) in terms of length, secondary structure, and conserved motifs (GNRA, C-rich) positioning (Fig. 6C), therefore, anticipating a similar interaction with tRNA_i_, highlighting a sequestering tendency by competing with cellular mRNAs.”

Rephrased sentence- “Like EMCV IRES, the type 1 IRES (Poliovirus, Coxsackie virus, etc.) also harbours the GNRA loop, preceded by a C-rich loop at its longest domain, known for long-range RNA-RNA interactions. The segment harbouring GNRA loop is highly conserved across the type 1 family of IRESs (Kim et al 2015). The domain I of EMCV IRES is similar to domain IV of polioviral IRES or other type 1 IRESs in terms of length, secondary structure, and conserved motifs (GNRA, C-rich) positioning (Fig. 6C). Therefore, we anticipate a similar interaction of domain IV (in type 1 IRES class) with tRNA_i_. Also, this interaction of IRES with tRNA_i_ could be a strategy by which these IRESs can sequester the tRNA_i_ pool in the cell, rendering them unavailable for capped cellular mRNAs.”

**Reviewer #3 (Recommendations for the authors):**

(1) For the revision process, the authors provided three atomic models alongside their corresponding cryo-EM density maps, including a 48S complex in closed conformation. Given this conformation, it is reasonable to interpret the structure as representing a post-start codon recognition state (late-stage initiation). However, this reviewer finds that the local resolution within the mRNA channel is insufficient to support the atomic model building as presented. The density does not allow for an unambiguous assignment of nucleotides in this region; the authors should either improve the local resolution or remove the modeled mRNA from the structure.

We understand the concern of the Reviewer. Although the mRNA density in the channel is poor, we modelled the mRNA with AUG-834 at the P site because the known biology of EMCV IRES. The EMCV IRES does not require scanning and directly positions the AUG-834 at the P site (Pestova et al 1996). In Pestova et al 1996, the intensity of the toeprint at AUG-834 is more intense than that of AUG-826. Further, AUG-834 lies in the Kozak context, whereas AUG-826 has a poor Kozak context, and AUG-826 codon is not in-frame with AUG-834. Therefore, the synthesis of the polypeptide requires AUG-834 at the P site. In our cryo-EM map, we observed that the tRNA_i_ is in a P_IN_ state, which indicates the recognition of the start codon, and we reasoned that it is very likely that AUG-834 is placed at the P site.

(2) As noted by the authors, the start codon in the EMCV IRES is positioned within a strong Kozak sequence. The nucleotide at position -3 is known to interact with eIF2α, yet, in the current model, A831 is positioned such that physical contact with eIF2α would be structurally impossible. This discrepancy raises concerns about the accuracy of the modeled eIF2α, which, like other regions of the structure, is not clearly supported by the cryo-EM density. The authors should revise the atomic model of eIF2α to ensure it is consistent with the experimental map and established molecular interactions.

In our analysis of EMCV IRES-48S PIC, we could observe eIF2α and eIF2γ in Map B and B1. However, the local resolution was low to model the entire protein with side-chains (Supplementary figure 1.2 A). So, we used rigid body fitting of eIF2α and eIF2γ (Author response image 6). From the model, we could trace the backbone of Arg55, however could not resolve the side chain. Similarly, the mRNA in the channel was modelled based on placement of AUG-834 at the P site for EMCV IRES, which enabled us to model the flanking residues, rather than at the nucleotide-level resolution. We anticipate that a higher resolution structure will be able to capture this interaction of eIF2α with mRNA nucleotide (-3), therefore refrained from commenting on this interaction in the manuscript. In the revised manuscript, we have removed the side chains of eIF2α and eIF2γ, and kept the Cα-backbone only. The map-model statistics of map B1 is updated in table 1.

**Author response image 6.** (left) Fitting of eIF2α model in the map. (right) Fitting of Cα backbone of eIF2α and mRNA in the map.

(3) The authors observed additional density interacting with ribosomal proteins uS19 and uS13, and tRNA, which they tentatively assign to domain I of the IRES. Although the local resolution in this region does not allow an unambiguous assignment, the interpretation is reasonable. However, further structural and functional validation is necessary to support this assignment. The authors should improve the local resolution, either by performing focused refinement or by increasing the number of particles used in the reconstruction.

The assignment of the extra density to domain I of the IRES was based on the architecture of the density. This density allows no other IRES domain to fit in this region (Supplementary figure 3.2). We tried to improve the local resolution using focused refinement, but the resolution was insufficient to resolve the IRES at the nucleotide level. Please see the above-mentioned comments in this regard on Pg 12.

(4) Figure 5 shows a slight shift in the position of the ternary complex. Is the observed tRNA conformation compatible with the structural rearrangements required for 60S subunit joining?

(5) In the discussion section, the authors state: "eIF3-eIF4G interaction is dispensable for EMCV IRES-48S PIC formation, so we do not rule out the possibility that EMCV IRES may dislodge eIF3 from its position on the solvent surface as observed in the case of HCV IRES (Hashem et al, 2013)." This statement is highly speculative. Is there any experimental or structural evidence to support this proposed mechanism in the context of EMCV IRES?

During the transition of 48S PIC to 80S elongation-competent complex, there are major changes in the conformation of tRNA_i_, due to the joining of eIF5B, and release of eIF2 (Petrychenko et al 2024). This joining event of eIF5B positions the tRNA_i_ elbow and acceptor stem towards the 40S body to aid 60S ribosomal subunit joining (Petrychenko et al 2024). However, in the context of EMCV IRES-48S PIC, we observed that the position of tRNA_i_ elbow and acceptor stem is towards the 40S head, and away from the body. On superimposing the human 48S PIC structure (before 60S joining), 48S-5 (PDB Id- 8PJ5- Petrychenko et al 2024), we note that tRNA_i_ in EMCV IRES-48S PIC is away from the canonical tRNA_i_ position (in contact with eIF5B). Therefore, we anticipate a change in tRNA_i_ conformation during eIF5B joining and eIF2 release. This hypothesis coincides with the fact that the IRES interacting with the tRNA_i_ elbow needs to be displaced from the position to facilitate the interaction of tRNA_i_ with eIF5B. Moreover, this rearrangement would also aid in 60S joining and prevent any clash with the IRES domain I. We have added this in Results selection 5 and Figure 5D.

Previous biochemical reports on the eIF3-eIF4G interaction suggested that eIF4G residues from 1011-1104 interact with eIF3 (Villa et al 2013). In the context of EMCV IRES, this region of eIF4G is not required to form 48S PIC on the IRES, suggesting the eIF3-eIF4G interaction is dispensable for EMCV IRES-48S PIC formation. However, the recent structure of the human canonical 48S PIC has shown that the eIF4G-HEAT1 domain can interact with eIF3 subunits c, h, and l, and that eIF4G-bound eIF4A can interact with 40S ribosomal protein eS7, thus mediating the interaction between eIF4-bound mRNA and the 43S PIC (Brito Querido et al 2024) but the known eIF3-binding region in eIF4G was not captured in the map. Although the canonical eIF3-eIF4G interaction is essential in the case of cap-dependent initiation, this interaction could be dispensable for 48S PIC formation on EMCV IRES. In case of HCV IRES-mediated initiation, eIF3 is displaced from its canonical position that facilitates the binding of HCV IRES to 40S ribosomal subunit (Hashem et al 2013). We did not see any density corresponding to eIF3 in the obtained maps. Further, we have used focused classification using a mask on the canonical eIF3 position; however, we do not see any density corresponding to eIF3 in the EMCV IRES-48S PIC complex. Therefore, we hypothesized the possibility that eIF3 might be dislodged from its canonical binding site on the 40S ribosomal subunit. However, as per the recent independent report on EMCV IRES-48S PIC, eIF3 is present in the complex (Bhattarcharjee et al 2025).

Hence, we have rephrased the existing sentence- “However, eIF3-eIF4G interaction is dispensable for EMCV IRES-48S PIC formation, so we do not rule out the possibility that EMCV IRES may dislodge eIF3 from its position on the solvent surface as observed in case of HCV IRES (Hashem et al 2013).”

Rephrased sentence- “However, the canonical eIF3-eIF4G interaction (Villa et al 2013) is dispensable for EMCV IRES-48S PIC formation (Lomakin et al 2000; Sweeney et al 2014), and we do not see any density for eIF3 even after focused classification. However, as per the recent independent report on reconstituted EMCV IRES-48S PIC, eIF3 is present in the complex at the canonical position (Bhattarcharjee et al 2025). This position of eIF3 further highlights the possibility that eIF4G-eIF4A proteins are also placed similarly to the canonical eIF3-eIF4G-eIF4A position (Brito Querido et al 2024) in context to EMCV IRES-48S PIC. Thus, placing eIF4G-domain J-K close to ES6 of 40S ribosome, which coincides with the previous hydroxyl radical cleavage assay (Yu et al 2011).”

(6) eIF4A has been shown to directly interact with eIF3 and facilitate recruitment of the 43S PIC. Does the interaction of the J-K domain with eIF4G/eIF4A, compatible with the known eIF4A-eIF3 interaction within the 43S PIC? In other words, during EMCV IRES-mediated initiation, could the eIF4A-eIF3 interaction functionally substitute for the eIF4G-eIF3 interaction?

Reports on EMCV IRES-mediated translation initiation have shown eIF4G as an essential component of 48S PIC formation (Pestova et al 1996; Lomakin et al 2000; Kolupaeva et al 2003; Sweeney et al 2014), where eIF4G directly interacts with domain J-K of IRES and eIF4A, thus enabling loading of eIF4A on the IRES. In our study, the cryo-EM map of EMCV IRES-48S PIC lacks density for eIF3 and eIF4 proteins, and locating eIF4F is challenging due to the inherent flexibility associated with the complex. Previous studies on EMCV IRES-48S PIC have mapped the location of eIF4G close to ES6 towards the platform side of the body and eIF3 using the hydroxyl radical cleavage assay (Yu et al 2011). The human 48S initiation complex structures have shown a similar location for eIF4G, which is at the mRNA exit site, contacting eIF3 (Brito Querido et al 2020; Brito Querido et al 2024). On overlapping the 18S rRNA of EMCV IRES-48S PIC to that of the human 48S PIC in closed conformation (PDB Id- 8OZ0), and further superimposing the J-K-St- eIF4G- eIF4A (PDB Id- 8HUJ) on human 48S PIC (PDB Id- 8OZ0) with respect to HEAT1 of eIF4G, the domain J-K becomes positioned at the subunit face of 40S body, close to ES6 (Author response image 7). This correlates with the previously reported position for eIF4G with respect to EMCV IRES-48S PIC (Yu et al 2011). The predicted model shows no clashes with the canonical eIF4A-eIF3/ eIF4G-eIF4A-eIF3 interaction, or with the domain J-K-eIF4G-eIF4A model. Thus, highlighting a possibly compatible interaction axis among eIF3-eIF4G-eIF4A-domain J-K of IRES.

**Author response image 7.** (upper left) Location of eIF4G-eIF4A in canonical human 48S PIC (PDB Id- 8OZ0). (upper right) Superimposition of 18S rRNA from human 48S and EMCV IRES 48S. (lower left) Superimposition of Human Closed 48S PIC structure (PDB Id- 8OZ0) on EMCV IRES-48S PIC model and placement of EMCV IRES- J-K domain-HEAT1-eIF4A structure (PDB Id- 8HUJ) with respect to eIF4G-HEAT1 domain. (lower right) Predicting location of eIF3 and eIF4 proteins in EMCV IRES-48S PIC.

(7) Assuming that the additional density near the ternary complex corresponds to Domain I of the IRES and that the codon in the P site represents the EMCV AUG start codon, what is the authors' mechanistic model for EMCV IRES-mediated initiation? Specifically, how is the mRNA positioned or inserted into the 40S mRNA channel in the absence of canonical scanning? As it stands, the discussion does not sufficiently address this key aspect of the EMCV initiation mechanism.

The EMCV IRES start codon (A-834) is directly placed in the P site (Pestova et al 1996), and the captured complex harboured the initiator tRNA in P_IN_ state with AUG at the P site. This start codon is preceded by domains J-K-L, where the J-K domain interacts with eIF4 proteins via eIF4G1-HEAT1 domain, and L domain is 20 residues upstream of the AUG and known to interact with eIF4B (Pestova et al 1996; de Quinto et al 2001). Based on the position and binding partners for these domains, the domain L could be placed at the mRNA exit site, preceded by domain J-K, which could be placed close to eIF4G-eIF4A position on EMCV IRES 48S PIC, near expansion segment 6 (ES6). The domain J-K can interact with eIF4G, localized close to the left foot or ES6 as per previous biochemical experiments (Yu et al 2011). This suggests that position of eIF4G and eIF4A could be the same as that of cap-dependent initiation where it can interact with eIF3 core subunits as well as the IRES domain J-K and the predicted path of mRNA from the exit site can follow the path of mRNA in human closed 48S PIC (PDB Id- 8OZ0), where it interacts with eIF3 core.

Examining the path of RNA in channel from the G-825 (exit site) to C-785 (domain J-K), we found the shortest distance is ~ 173 Å. This bridge could be filled by a single-stranded stretch of 40 nucleotides. However, the presence of domain L (stem loop- residues- 782 to 810) might hinder the placement of A-834 in the P-site (Author response image 8). We anticipate that to accommodate the start codon at the P site, either the domain L stem loop is resolved, which is an energetically expensive process (free energy of the thermodynamic ensemble is -11.12 kcal/mol, predicted using RNAfold). Another way could be a change in the orientation or conformation of domain J-K such that the start codon is directly placed at the P site without resolving domain L.

**Author response image 8.** (left) The shortest distance between the last fitted residue- 825th of EMCV IRES to 785th of J-K domain of IRES (keeping eIF4G position same as that of PDB Id- 8OZ0) is 173 Å. (right) Tracing the path of mRNA (red) upstream of AUG coming out of the exit site of 40S ribosome and the possible position of eIF4G on EMCV IRES-48S PIC. Addition of nucleotides between C-785 and G-825 would fill the gap. The route of predicted mRNA from the exit channel is based on the mRNA (green) exiting the channel (PDB Id- 8OZ0).

The domain I is followed by domain J-K, close to the left foot of the 40S ribosomal subunit as per previous biochemical experiments (Yu et al 2011). However, the minimum distance connecting the I domain at 601st nucleotide to 682nd nucleotide of domain J-K (at the predicted location) is ~300 Å, which might be difficult to be covered by 80 nucleotides (from 601 to 682), present as a double helical strand. We suppose there could be instances of J-K domain repositioning in the EMCV IRES-48S PIC such that the I domain apical region can contact the 40S head and simultaneously place the start codon at the P site (Author response image 9).

**Author response image 9.** Rotated views of EMCV IRES domains- I apical part in contact with 40S head and tRNAi and predicted location of J-K domain in contact with eIF4G, close to the left foot of 40S (predicted from PDB Id- 8OZ0). The minimum distance connecting 601st nucleotide in I domain to 682nd nucleotide in J-K domain is 295.5 Å.

We lack any details on the other IRES domains, such as domain I lower stem, domain J-K, or L; therefore, we refrained from commenting on these in our manuscript.

(8) Supplementary Figure 1 is missing labels for the RNA ladders.

The size of the DNA ladder used is mentioned.

References:

Bhattacharjee S, Abaeva IS, Brown ZP, Arhab Y, Fallah H, Hellen CUT, Frank J, Pestova TV. The mechanism of ribosomal recruitment during translation initiation on Type 2 IRESs. bioRxiv [Preprint]. 2025 Jun 11:2025.06.11.659010. doi: 10.1101/2025.06.11.659010. PMID: 40568087; PMCID: PMC12191231.

Brito Querido J, Sokabe M, Díaz-López I, Gordiyenko Y, Fraser CS, Ramakrishnan V. The structure of a human translation initiation complex reveals two independent roles for the helicase eIF4A. Nat Struct Mol Biol. 2024 Mar;31(3):455-464. doi: 10.1038/s41594-023-01196-0. Epub 2024 Jan 29. PMID: 38287194; PMCID: PMC10948362.

Brito Querido J, Sokabe M, Kraatz S, Gordiyenko Y, Skehel JM, Fraser CS, Ramakrishnan V. Structure of a human 48S translational initiation complex. Science. 2020 Sep 4;369(6508):1220-1227. doi: 10.1126/science.aba4904. PMID: 32883864; PMCID: PMC7116333.

Chamond N, Deforges J, Ulryck N, Sargueil B. 40S recruitment in the absence of eIF4G/4A by EMCV IRES refines the model for translation initiation on the archetype of Type II IRESs. Nucleic Acids Res. 2014;42(16):10373-84. doi: 10.1093/nar/gku720. Epub 2014 Aug 26. PMID: 25159618; PMCID: PMC4176346.

Dorn G, Gmeiner C, de Vries T, Dedic E, Novakovic M, Damberger FF, Maris C, Finol E, Sarnowski CP, Kohlbrecher J, Welsh TJ, Bolisetty S, Mezzenga R, Aebersold R, Leitner A, Yulikov M, Jeschke G, Allain FH. Integrative solution structure of PTBP1-IRES complex reveals strong compaction and ordering with residual conformational flexibility. Nat Commun. 2023 Oct 13;14(1):6429. doi: 10.1038/s41467-023-42012-z. PMID: 37833274; PMCID: PMC10576089.

Duke GM, Hoffman MA, Palmenberg AC. Sequence and structural elements that contribute to efficient encephalomyocarditis virus RNA translation. J Virol. 1992 Mar;66(3):1602-9. doi: 10.1128/JVI.66.3.1602-1609.1992. PMID: 1310768; PMCID: PMC240893.

Fernández N, Fernandez-Miragall O, Ramajo J, García-Sacristán A, Bellora N, Eyras E, Briones C, Martínez-Salas E. Structural basis for the biological relevance of the invariant apical stem in IRES-mediated translation. Nucleic Acids Res. 2011 Oct;39(19):8572-85. doi: 10.1093/nar/gkr560. Epub 2011 Jul 8. PMID: 21742761; PMCID: PMC3201876.

Hashem Y, des Georges A, Dhote V, Langlois R, Liao HY, Grassucci RA, Pestova TV, Hellen CU, Frank J. Hepatitis-C-virus-like internal ribosome entry sites displace eIF3 to gain access to the 40S subunit. Nature. 2013 Nov 28;503(7477):539-43. doi: 10.1038/nature12658. Epub 2013 Nov 3. PMID: 24185006; PMCID: PMC4106463.

Imai S, Suzuki H, Fujiyoshi Y, Shimada I. Dynamically regulated two-site interaction of viral RNA to capture host translation initiation factor. Nat Commun. 2023 Aug 28;14(1):4977. doi: 10.1038/s41467-023-40582-6. PMID: 37640715; PMCID: PMC10462655.

Kim H, Kim K, Kwon T, Kim DW, Kim SS, Kim YJ. Secondary structure conservation of the stem-loop IV sub-domain of internal ribosomal entry sites in human rhinovirus clinical isolates. Int J Infect Dis. 2015 Dec;41:21-8. doi: 10.1016/j.ijid.2015.10.015. Epub 2015 Oct 27. PMID: 26518063.

Lomakin IB, Hellen CU, Pestova TV. Physical association of eukaryotic initiation factor 4G (eIF4G) with eIF4A strongly enhances binding of eIF4G to the internal ribosomal entry site of encephalomyocarditis virus and is required for internal initiation of translation. Mol Cell Biol. 2000 Aug;20(16):6019-29. doi: 10.1128/mcb.20.16.6019-6029.2000. PMID: 10913184; PMCID: PMC86078.

López de Quinto S, Martínez-Salas E. Conserved structural motifs located in distal loops of aphthovirus internal ribosome entry site domain 3 are required for internal initiation of translation. J Virol. 1997 May;71(5):4171-5. doi: 10.1128/JVI.71.5.4171-4175.1997. PMID: 9094703; PMCID: PMC191578.

Lozano G, Francisco-Velilla R, Martinez-Salas E. Ribosome-dependent conformational flexibility changes and RNA dynamics of IRES domains revealed by differential SHAPE. Sci Rep. 2018 Apr 3;8(1):5545. doi: 10.1038/s41598-018-23845-x. PMID: 29615727; PMCID: PMC5882922.

Maloney A, Joseph S. Validating the EMCV IRES Secondary Structure with Structure-Function Analysis. Biochemistry. 2024 Jan 2;63(1):107-115. doi: 10.1021/acs.biochem.3c00579. Epub 2023 Dec 11. PMID: 38081770; PMCID: PMC10896073.

Pestova TV, Hellen CU, Shatsky IN. Canonical eukaryotic initiation factors determine initiation of translation by internal ribosomal entry. Mol Cell Biol. 1996 Dec;16(12):6859-69. doi: 10.1128/MCB.16.12.6859. PMID: 8943341; PMCID: PMC231689.

Petrychenko V, Yi SH, Liedtke D, Peng BZ, Rodnina MV, Fischer N. Structural basis for translational control by the human 48S initiation complex. Nat Struct Mol Biol. 2024 Sep 17. doi: 10.1038/s41594-024-01378-4. Epub ahead of print. PMID: 39289545.

Roberts LO, Belsham GJ. Complementation of defective picornavirus internal ribosome entry site (IRES) elements by the coexpression of fragments of the IRES. Virology. 1997 Jan 6;227(1):53-62. doi: 10.1006/viro.1996.8312. PMID: 9007058.

Robertson ME, Seamons RA, Belsham GJ. A selection system for functional internal ribosome entry site (IRES) elements: analysis of the requirement for a conserved GNRA tetraloop in the encephalomyocarditis virus IRES. RNA. 1999 Sep;5(9):1167-79. doi: 10.1017/s1355838299990301. PMID: 10496218; PMCID: PMC1369840.

Sweeney TR, Abaeva IS, Pestova TV, Hellen CU. The mechanism of translation initiation on Type 1 picornavirus IRESs. EMBO J. 2014 Jan 7;33(1):76-92. doi: 10.1002/embj.201386124. Epub 2013 Dec 15. PMID: 24357634; PMCID: PMC3990684.

Velazquez MA, Nuthalapati SS, Hankinson J, Fominykh K, Lulla V, Sweeney TR, Hill CH. Structural and mechanistic insights into translation initiation on the enterovirus Type 1 IRES. bioRxiv [Preprint]. 2025 Oct 3: 2025.10.04.680434. doi: 10.1101/2025.10.04.680434.

Yu Y, Sweeney TR, Kafasla P, Jackson RJ, Pestova TV, Hellen CU. The mechanism of translation initiation on Aichivirus RNA mediated by a novel type of picornavirus IRES. EMBO J. 2011 Aug 26;30(21):4423-36. doi: 10.1038/emboj.2011.306. PMID: 21873976; PMCID: PMC3230369.
